# Early Versus Late Wake‐Up Call After Out‐Of‐Hospital Cardiac Arrest: Protocol for a Multicenter Randomized Comparison Within the Danish Out‐of‐Hospital Cardiac Arrest (DANOHCA) Trial

**DOI:** 10.1111/aas.70272

**Published:** 2026-06-07

**Authors:** Anders Morten Grejs, Jesper Kjaergaard, Jo Bønding Andreasen, Bodil Steen Rasmussen, Christian Hassager, Martin Abild Stengaard Meyer, Lars Peter Kloster Andersen, Henrik Schmidt, Simon Moelstroem, Sören Möller, Christhopher Torp, Steffen Christensen, Jacob Eifer Moeller

**Affiliations:** ^1^ Department of Intensive Care Medicine Aarhus University Hospital Aarhus Denmark; ^2^ Department of Clinical Medicine Aarhus University Aarhus Denmark; ^3^ Department of Cardiology Copenhagen University Hospital‐Rigshospitalet Copenhagen Denmark; ^4^ Department of Clinical Medicine University of Copenhagen Copenhagen Denmark; ^5^ Department of Anaesthesiology and Intensive Care Aalborg University Hospital Aalborg Denmark; ^6^ Department of Clinical Medicine Aalborg University Aalborg Denmark; ^7^ Department of Anaesthesiology Zealand University Hospital Koege Denmark; ^8^ Department of Anaesthesiology and Intensive Care Odense University Hospital Odense Denmark; ^9^ Department of Clinical Medicine Southern Denmark University Odense Denmark; ^10^ Department of Cardiothoracic Anaesthesiology Rigshospitalet, Copenhagen University Hospital Copenhagen Denmark; ^11^ Epidemiology, Biostatistics and Biodemography, Department of Public Health University of Southern Denmark Odense Denmark

**Keywords:** out‐of‐hospital cardiac arrest, post cardiac arrest care, post resuscitation, sedation, wake‐up call

## Abstract

**Background:**

Survivors of out‐of‐hospital cardiac arrest (OHCA) who remain comatose after return of spontaneous circulation are routinely sedated and mechanically ventilated during early post‐resuscitation care. Although prolonged sedation has traditionally been considered necessary, contemporary normothermia‐based temperature control allows earlier wake‐up call. The optimal timing of an early wake‐up call remains unknown and may influence overall mortality, neurological recovery, duration of mechanical ventilation, and length of hospital stay.

**Methods:**

The Danish Out‐of‐Hospital Cardiac Arrest (DANOHCA) trial (clinicaltrials.gov identifier: NCT05895838; and euclinicaltrials.eu, identifier: 2024–515,997–28‐00) is an investigator‐initiated, multicenter, randomized clinical trial using a 2 × 2 × 2 × 2 factorial design evaluating four interventions in patients resuscitated from OHCA. The present protocol describes the comparison of an early versus late wake‐up call strategy. Adult patients (≥ 18 years) with presumed cardiac‐cause of OHCA, sustained return of spontaneous circulation, and persistent unconsciousness on intensive care unit admission are randomized 1:1 to early wake‐up call (≤ 6 h after randomization) or late wake‐up call (28–36 h after randomization). Wake‐up call includes interruption of sedation, assessment of neurology, and may be followed by extubation if predefined neurological and respiratory criteria are fulfilled. The primary endpoint is days alive and out of hospital within 30 days after randomization. Analyses will follow a modified intention‐to‐treat principle.

**Perspectives:**

Optimizing post‐resuscitation care remains a cornerstone in managing comatose cardiac arrest survivors and improving outcomes. We hypothesize that sedation for 28–36 h leads to more days alive outside of the hospital in 30 days compared to sedation for ≤ 6 h.

**Trial Registration:** EudraCT number: 2016‐003265‐26; EU CTIS no 2024‐515997‐28‐00; ClinicalTrials.gov identifier: NCT05895838.

## 1 Introduction

Out‐of‐hospital cardiac arrest (OHCA) remains a leading cause of death and neurological disability, despite substantial improvements in prehospital care and post‐resuscitation management [1, 2, 3]. Among patients admitted to the intensive care unit (ICU) in a comatose state after sustained return of spontaneous circulation (ROSC), in‐hospital mortality remains high and is most often related to hypoxic–ischemic brain injury [4, 5, 6, 7, 8].

Early post‐cardiac arrest care aims to mitigate secondary brain injury while allowing sufficient time for neurological recovery before prognostication. Sedation and mechanical ventilation have traditionally been integral components of this phase, largely to facilitate hypothermic temperature and shivering control [4]. However, in general ICU patients, prolonged sedation is associated with well‐documented adverse effects, including delayed awakening, protracted mechanical ventilation, and extended ICU and hospital stay [9, 10, 11, 12, 13].

Despite the central role of sedation in post‐cardiac arrest care, evidence guiding the optimal timing of sedation interruption and wake‐up call is sparse. Recent studies found that an early wake‐up call was feasible in comatose OHCA patients and hence challenged the necessity of prolonged deep sedation [14, 15, 16]. However, in the Danish NONSEDA trial, a strategy of no continuous sedation compared with light sedation in mechanically ventilated patients with respiratory failure was associated with a 5.4% higher mortality in the non‐sedation group, although not statistically significant [17]. The Targeted Temperature Management 2 (TTM2) trial demonstrated no benefit of hypothermia at 33°C compared with active normothermia with fever prevention [18]. As normothermia can generally be achieved without deep sedation, this paradigm shift has highlighted an unresolved and clinically important question: whether routine sedation and mechanical ventilation for 24 h or longer remain justified, or whether earlier awakening is feasible and safe in patients managed with up‐to‐date post‐resuscitation care [4]. Importantly, the timing of a wake‐up call may directly influence duration of mechanical ventilation, neurological assessment, delirium incidence, and length of hospitalization—outcomes of high relevance to patients, relatives, and healthcare systems.

The Danish Out‐of‐Hospital Cardiac Arrest (DANOHCA) trial is an investigator‐initiated, multicenter randomized controlled trial using a factorial design to evaluate four modifiable components of early post‐cardiac arrest care. The present protocol describes the early versus late wake‐up call comparison within DANOHCA. Patients are randomized to early sedation interruption and assessment for extubation within 6 h after randomization, or to a delayed wake‐up call at 28–36 h after randomization. The primary objective is to identify the wake‐up call strategy that optimizes patient outcomes while maintaining patient safety [19]. We hypothesize that sedation for 28–36 h leads to more days alive outside of the hospital in 30 days compared to sedation for ≤ 6 h.

## 2 Methods

### 2.1 Trial Design

The study is an investigator‐initiated, randomized, controlled, multicenter clinical trial with co‐enrolment into four different interventions in a 2 × 2 × 2 × 2 factorial design. The pharmacological interventions will be placebo‐controlled, double‐blinded interventions and physiological interventions of early wake‐up call as well as the back‐rest position interventions open label. Following successful completion of screening procedures, patients will be randomized in a 1:1 fashion to receive either the active intervention or placebo/control for each intervention arm. The randomization will be revealed by opening a sealed box containing the study drug vials/tablets, instructions for wake‐up call and extubation and backrest positioning and general information and link/QR codes for data entry into the electronic case report form (eCRF).

The DANOHCA trial is registered at clinicaltrials.gov (identifier: NCT05895838) and at the EU CTIS under 2024‐515997–28‐00 accessible at euclincaltrials.eu (previously registered with EudraCT number: 2021‐005876‐21 at clinicaltrialsregister.eu). The trial has been approved by the Danish Medicines Agency (DKMA) and Danish Medical Research Ethics Committees in accordance with the Clinical Trials Regulation No 536/2014 and registered with CTIS Trial number 2024‐515997‐28‐00; Transition authorized with conditions on 11‐10‐2024; the current protocol v02‐12‐2025 (SM‐2) was authorized on 05‐12‐2025. Prior to the transition to CTR the trial had been approved by the Danish Medicines Agency no. 2021121966 since 07‐02‐2022 and the regional ethics committee of the Capital Region journal number H‐21077461 since 08‐07‐2022.

Patients and the public were not directly involved in the design of the trial due to the emergency nature of OHCA and the inability of patients to participate in trial planning. The choice of primary and secondary outcomes was informed by patient‐centered considerations, including survival and time spent outside hospital, which are highly relevant to patients and relatives.

### 2.2 Trial Conduct

The trial will be conducted according to all applicable national and international legislation and guidelines including the revised Declaration of Helsinki [17] and the international Good Clinical Practice guidelines [18]. The trial and the protocol are developed in agreement with the International Conference on Harmonization (ICH) guidelines [18, 19, 20] and the Standard Protocol Items: Recommendations for Interventional Trials (SPIRIT) statement [21, 22]. Monitoring activities include verification of consent procedures, source data verification, adherence to the approved protocol, and compliance with applicable regulatory requirements. Monitoring is performed according to a predefined monitoring plan.

The trial adheres to EU and national legislation on medical research in patients in emergency situations being temporarily incapacitated and unable to provide informed consent. National legislation requires proxy consent from a trial guardian, and as soon as possible following admission, consent from next of kin. If the patient regains consciousness he/she will have to consent themselves. Participants or their legal representatives may withdraw consent at any time without consequences for ongoing or future medical care. Data collected prior to withdrawal are retained and analyzed in accordance with applicable regulations unless consent for data use is explicitly withdrawn.

### 2.3 Trial Interventions

**FIGURE 1:**
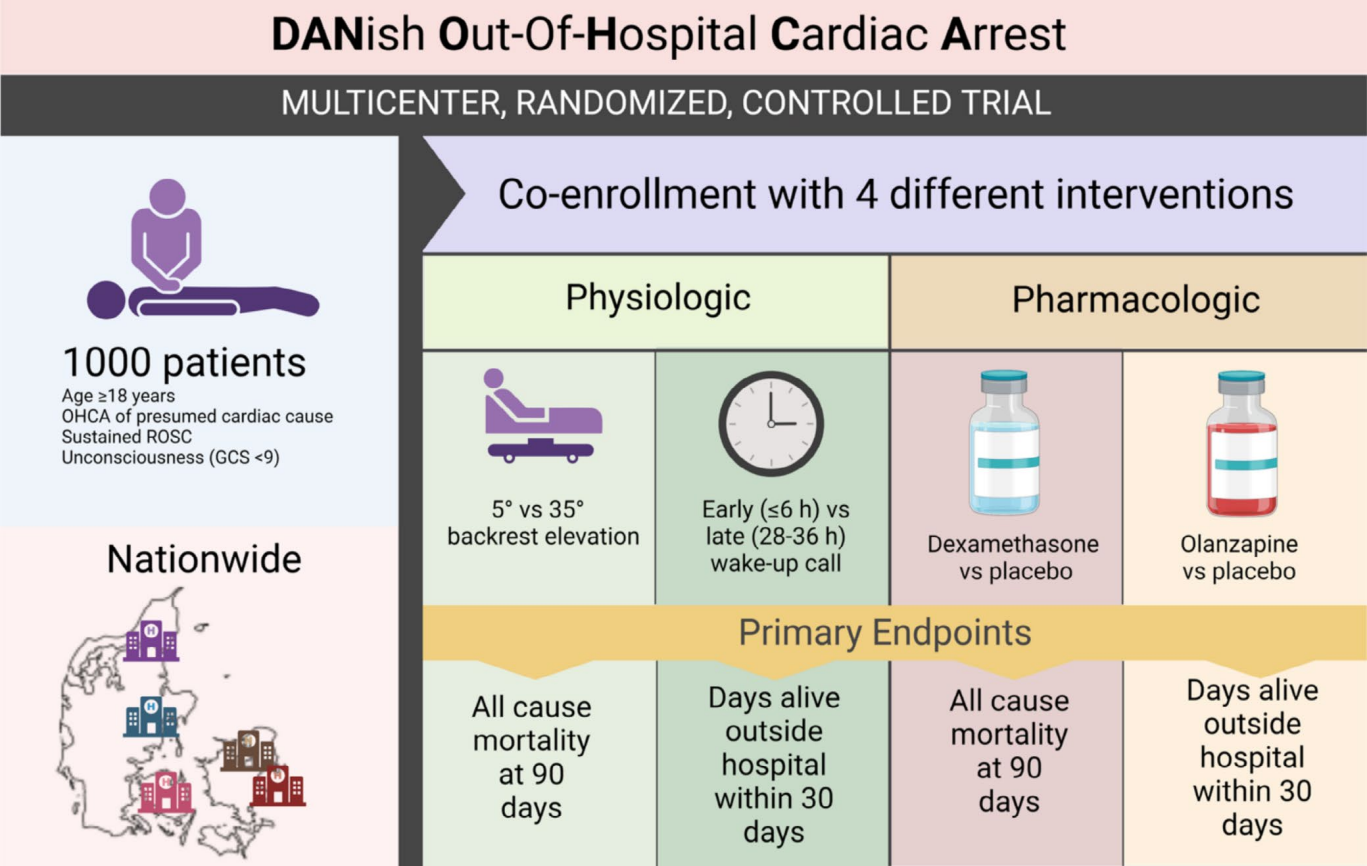
DANOHCA Overview: Overview of the total DANOHCA trial with the 2 × 2 × 2 × 2 factorial design, details of the two physiologic and the two pharmacologic interventions, and site location.

By randomization, patients in the DANOHCA trial will be allocated 1:1 to either the active intervention or placebo/control intervention for each of four interventions (Figure 1). The two pharmacological interventions will be 20 mg of dexamethasone phosphate intravenously at randomization and the following two mornings for a total of three doses, and 10 mg olanzapine or placebo tablet at randomization and the following two evenings for a total of three doses. The two physiological interventions consist of backrest elevation to 35° vs. 5° during the initial period of sedation and ventilation, as well as an early wake‐up call and potential extubation ≤ 6 h or a late wake‐up call and extubation between 28–36 h after randomization.

Balance of allocation into the 16 possible combinations of allocation is monitored when generating the randomization sequence and as part of interim analyses.

The wake‐up call intervention compares two predefined strategies for interruption of sedation timed in relation to randomization. Patients allocated to the early wake‐up call undergo sedation interruption and clinical assessment for extubation within 6 h after randomization. Patients allocated to the late wake‐up call are maintained at a sedation level of RASS −5 and are evaluated for a wake‐up call and extubation 28–36 h after randomization.

Prior to the scheduled wake‐up call, sedation is permitted in the early wake‐up call group as clinically indicated and is mandatory in the late wake‐up call group, unless contraindicated for safety reasons. Sedation practices, including choice of agents, adhere to local ICU standards and are recorded in the eCRF.

At the time of the wake‐up call, all continuous infusions of sedatives and opioids are interrupted. Patients are neurologically assessed by Glasgow Coma Score, RASS and ability to follow commands. A successful wake‐up call is defined as GCS ≥ 12/RASS 0–1, able to raise arm or voluntary handshake on command. If wake‐up call is successful patients are assessed clinically for readiness for extubation using predefined respiratory, and hemodynamic criteria. Definition for “ready for extubation,” is positive spontaneous breathing trial and low ventilator settings (pressure support ≤ 14, PEEP ≤ 8 (10 if obese), and FiO2 ≤ 40%). A wake‐up call may for both groups be aborted for the following reasons: seizures, respiratory distress, shock, or “other cause” with specification. Stable hemodynamic status is defined as no need for escalating vasopressor support.

If extubation criteria are fulfilled, extubation is performed according to local ICU procedures. If extubation criteria are not fulfilled, sedation may be resumed at the discretion of the treating clinician and further attempts at awakening and extubation are conducted according to standard clinical practice. Reasons for not performing extubation at the scheduled wake‐up call are documented in the eCRF.

The intervention is pragmatic and embedded in routine intensive care. Treating clinicians may deviate from the protocol at any time if required for patient safety. All protocol deviations related to the wake‐up call intervention are prospectively recorded.

A successful wake‐up call and extubation will terminate the Backrest elevation protocol. However, the dexamethasone/placebo and olanzapine/placebo protocols are continued for three administrations or until the patient leaves the ICU.

#### 2.3.1 Changing Sedation

Sedation may be resumed after the wake‐up call at the discretion of the treating clinician if extubation is not performed or if clinically indicated. No protocolized sedation depth targets are mandated after the wake‐up call.

All sedation‐related data, including agents used, duration of sedation, timing of sedation interruption, and reasons for deviation from the assigned strategy, are prospectively recorded in the eCRF.

#### 2.3.2 General Intensive Care Management

All patients receive standard intensive care according to European post‐resuscitation guidelines [4] and local institutional protocols. This includes hemodynamic monitoring and support, mechanical ventilation, glucose control, electrolyte management, and prevention of secondary organ injury.

Temperature control follows a normothermia‐based strategy with active prevention of fever. Shivering is managed according to local practice and may include non‐pharmacological measures and pharmacological agents at the discretion of the treating clinicians. The use of neuromuscular blocking agents is permitted if required for severe respiratory failure, refractory shivering, or severe ventilator asynchrony and is recorded in the eCRF. If these agents are used, a wake‐up call must not be performed before any confounding is considered and excluded.

Concomitant treatments, including vasoactive medications, ventilatory settings, and temperature control interventions, are not protocolized beyond the wake‐up call intervention and are documented prospectively in the eCRF. Neuroprognostication and decisions regarding withdrawal of life‐sustaining therapy are deferred until at least 72 h after admission and are performed according to guidelines, independent of trial allocation [4, 20].

### 2.4 Blinding

The wake‐up call intervention is open‐label, as blinding of sedation interruption and timing of extubation is not feasible. Treating clinicians, bedside staff, and patients are therefore aware of allocation to early or late wake‐up call. Outcome assessors for outcomes derived from registry data and follow‐up assessments are blinded to allocation whenever feasible. Statistical analyses will be conducted according to a prespecified analysis plan, with blinding maintained for treatment allocation until the database is unlocked.

### 2.5 Patient Eligibility and Enrollment

The study is coordinated by The Department of Cardiac Intensive Care, Copenhagen University Hospital—Rigshospitalet. The study will enroll consecutive patients from all 5 ICUs in Denmark where comatose resuscitated OHCA patients with a presumed cardiac cause are admitted: Copenhagen University Hospital, Aalborg University Hospital, Aarhus University Hospital, Zealand University Hospital, and Odense University Hospital.

All OHCA patients admitted to one of the five ICUs will be entered in a screening log, either by part of the inclusion and randomization or post hoc. The screening log includes the patient's ID, demographic data, and reasons for not including the patient in the trial (lack of inclusion criteria, presence of exclusion criteria, logistical reasons—in that order). If all inclusion criteria are fulfilled and no exclusion criteria are found (Table 1), the first trial guardian is contacted by phone for approval. A positive response will unlock further inclusion in the trial and provide a trial‐specific patient identifier. As soon as possible after admittance to the hospital, the patient will be evaluated and randomized. Following inclusion, a sealed cardboard box with a trial kit will be opened by the study personnel at the ICU. A QR code will link to a REDCap survey in which the trial patient identifier, CPR number, and Kit ID are linked.

**TABLE 1:** Inclusion and exclusion criteria.

Inclusion criteria	Exclusion criteria
All the listed criteria (1–4) must be met. 1. Age ≥ 18 years 2. OHCA of presumed cardiac cause 3. Sustained ROSC^a^ 4. Unconsciousness (GCS < 9) (patients not able to obey verbal commands) after sustained ROSC at the time of randomization	1. Females of childbearing potential if pregnancy is suspected (unless a negative HCG test can rule out pregnancy within the inclusion window) 2. Known bleeding diathesis (medically induced coagulopathy (e.g., warfarin, NOAC, clopidogrel) does not exclude the patient). 3. Suspected or confirmed acute intracranial bleeding 4. Suspected or confirmed acute stroke 5. Unwitnessed asystole 6. Known limitations in therapy and Do Not Resuscitate‐order 7. Known disease making 180 days survival unlikely 8. Known pre‐arrest Cerebral Performance Category (CPC) 3 or 4 functional status 9. > 3 h (180 min) from ROSC to screening 10. Systolic blood pressure < 80 mmHg despite fluid loading/vasopressor and/or inotropic medication^b^ 11. Use of intra‐aortic balloon pump/axial flow device/ECMO^c^ 12. Temperature on admission < 30°C. 13. Known allergy for dexamethasone or olanzapine 14. Ongoing (within 48 h) treatment with olanzapine or dexamethasone 15. Known back or hip condition that precluded the patients from being positioned with backrest from 0 to 45‐degree angle 16. Known or suspected Long QT Syndrome (LQTS) 17. Known active fungal disease. Localized skin lesions do not exclude patients from inclusion. 18. Estimated body weight < 45 kg.

*Note:* Since post resuscitation care and intervention are regarded as emergency procedures and should not be delayed, there should only be taken easily accessible sources (previous in‐house chart records, history from emergency medical services) to assess criteria 2, 5, 6, 7, 8, 12, 13, 14, and 15.

^a^ Sustained ROSC: Sustained ROSC is when chest compressions or mechanical circulatory support have not been required for 20 consecutive minutes and signs of circulation persist.

^b^ If the systolic blood pressure (SBP) is recovering during the inclusion window (180 min) the patient may be included.

^c^ If the patient is weaned and the device is removed during the inclusion window (180 min) the patient may be included.

Outcome of patients fulfilling inclusion criteria will be assessed by coupling the CPR registry at the end of the trials and patient IDs will be deleted.

Co‐enrollment into further trials must be permitted by the steering group following careful evaluation of the risk of interaction with the four per‐protocol interventions.

### 2.6 Baseline Characteristics and Endpoints

The baseline characteristics are summarized in Table 2 and the primary and secondary endpoints are summarized in Table 3.

**TABLE 2:** Baseline characteristics.

Characteristic	Total (*N*=)	Early WUC (*N*=)	Standard sedation (*N*=)
Demographic characteristics			
Age—yr			
Male sex—no. (%)			
Cardiac arrest characteristics			
Witnessed arrest—no. (%)			
Bystander cardiopulmonary resuscitation—no. (%)			
Initial rhythm—no. (%)			
Shockable (ventricular fibrillation or pulseless ventricular tachycardia)			
Nonshockable (pulseless electrical activity or asystole)			
Presumed cardiac cause—no. (%)			
Arrest at home—no. (%)			
Time from collapse to return of spontaneous circulation—min			
Total dose of epinephrine—mg			
Mechanical CPR used—no. (%)			
Clinical status on admission			
Lactate—mmol per liter			
Arterial pH			
Base excess—mmol per liter			
PaO_2_—kPa/mmHg			
PaCO_2_—kPa/mmHg			
Glasgow Coma Scale motor score			
Bilaterally absent pupillary light reflex—no. (%)			
Shock on admission^a^—no. (%)			
Mean arterial pressure—mm Hg			
Vasopressor use—no. (%)			
Coexisting conditions			
Hypertension—no. (%)			
Myocardial infarction—no. (%)			
Heart failure—no. (%)			
Diabetes mellitus—no. (%)			
Chronic kidney disease—no. (%)			
Chronic obstructive pulmonary disease—no. (%)			
Coronary angiography and PCI			
Immediate coronary angiography—no. (%)			
Percutaneous coronary intervention—no. (%)			

*Note:* Data are presented as means ± SD, medians with interquartile ranges, or numbers and percentages.

^a^ Shock was defined as the need for vasopressor support or a mean blood pressure of less than 60 mmHg.

**TABLE 3:** Primary and secondary endpoints overview.

	Total (*N*=)	Early WUC (*N*=)	Late WUC (*N*=)
Primary endpoint			
Days alive outside of hospital within 30 days			
Secondary endpoints			
All‐cause mortality at 90 days			
CPC and mRS at 3–6 months follow‐up			
Duration of mechanical ventilation			
ICU length of stay			
Hospital length of stay			

Abbreviations: CPC, cerebral performance category score; ICU, intensive care unit; mRS, modified Rankin Scale; WUC, wake‐up call.

#### 2.6.1 Primary Endpoint

The primary endpoint for the study is days alive and out of the hospital within 30 days in the two treatment arms, as a modified intention to treat analysis. The primary endpoint is determined as follows: A day (≥ 24 h) spent at health care facility is counted as time in hospital. Outpatient visits and brief hospitalizations for less than 24 h are not counted as days in hospital. Days admitted to hospital or as deceased within 30 days from randomization are subtracted from 30 days and the result is rounded down to the nearest whole number of days. For patients dying during the initial 30 days, the time spent alive outside of hospital is retained for the endpoint calculation.

#### 2.6.2 Secondary Endpoints

Secondary endpoints are as follows:

- All‐cause mortality at 90 days.
- Cerebral Performance Category (CPC) and the modified Rankin Scale (mRS) at 3–6 months.
- Duration of mechanical ventilation.
- ICU length of stay.
- Hospital length of stay.

The 3–6 months CPC and mRS scores are collected during routine trial follow‐up, conducted either through physical attendance or telephone interview according to participant preference. In either case, patients are asked to complete a questionnaire in advance. Duration of mechanical ventilation, ICU‐ and hospital length of stay account only for the primary admission.

#### 2.6.3 Exploratory Endpoints

Explorative endpoints are as follows:

- Duration of sedation
  ○ Reason for continued sedation: neurological, respiratory, hemodynamic
- Biomarkers of neurological injury include serum neuron‐specific enolase measured 48 and 72 h after randomization [21].
- Proportion of unconscious patients at 96 h.
- CPC and mRS at intensive unit discharge and hospital discharge.
- Markers of cardiac and kidney injury‡:
  ○ TNT or TNI and CKMB (cardiac) and proBNP during initial 72 h
  ○ Creatinine during initial 72 h and the use of dialysis during the first 30 days post OHCA (renal)
- Delirium‐related outcomes assessed using the Confusion Assessment Method for the ICU [12, 22], including.
  ○ Number of CAM‐ICU positive patients 48 h after randomization
- Neurological and functional outcomes are assessed using the Cerebral Performance Category and the modified Rankin Scale at ICU discharge, at hospital discharge [23, 24].

‡The rationale for comparing cardiac and renal biomarkers between sedation groups is based on the potential physiological effects of sedation depth on hemodynamics and stress responses. Deeper sedation may reduce sympathetic tone and blood pressure, potentially affecting organ perfusion, whereas lighter sedation may increase sympathetic activation and myocardial oxygen demand.

### 2.7 Statistical Methods

#### 2.7.1 Sample Size Considerations for the Wake‐Up Call Intervention

We estimate that 35% will not survive until hospital discharge (0 days alive out of hospital), and the expected mean number of days out of hospital for survivors is 15 days. Therefore, the mean number of days outside hospital will be 9.8 days for the population with an estimated standard deviation of 7 days. To detect an increase of 1.5 hospital free days with power > 90% and alfa 5%, a total of 920 patients will be required.

#### 2.7.2 Statistical Analyses

The data will be analyzed and reported as four individual trials, hence no adjustment for multiple comparison is planned for the primary endpoint. This is justified because almost all previous trials involving the hospital phase of OHCA patients have been neutral; therefore, all four interventions are accepted without considering potential interaction. However, should more than one of the interventions be non‐neutral, a post hoc evaluation of interactions between interventions will be performed in the primary regression model. Interactions are considered significant if the interaction term has *p* < 0.01 and demonstrate a clinically important effect defined as risk reduction of ≥ 5.6%. This will be highlighted in the reporting of the results of the primary outcome along with stratification by the interacting intervention allocation. Also, as an investigation of potential safety aspects related to possible interactions between the four interventions, we will report on interactions related to mortality and occurrence of other serious adverse events.

All analyses will be conducted according to the modified intention‐to‐treat (ITT) population. Modified ITT in this regard means all patients included except those where consent could not be obtained. The primary endpoint analysis will include the modified ITT population, and as a sensitivity analysis we will conduct a per protocol population to assess the effect, including only those trial participants who have adhered to the assigned sedation regimen.

The analyses are two‐sided with a significance level of 0.05. Throughout, categorical variables will be presented as counts with proportions, whereas continuous variables will be presented as mean ± SD if normally distributed, and as median (25th percentile–75th percentile) if non‐normally distributed, as evaluated by quantile‐quantile plots. Any changes from the pre‐specified analysis plan will be reported.

Prespecified subgroup analyses contain sex, age, initial rhythm, time to ROSC, lactate level upon arrival, and witnessed versus unwitnessed/bystander versus non‐bystander CPR.

#### 2.7.3 Analysis of Continuous, Categorical and Ordinal Endpoints

Continuous endpoints including the primary outcome of days alive out of the hospital within 30 days will be analyzed by application of Wilcoxon rank‐sum test (Mann–Whitney *U* test) between the two treatment arms and reported as relative difference with bootstrapped 95% confidence intervals (CI). As a pre‐specified supplementary analysis, we will also express the primary outcome as mean between‐group difference with 95% CI. To test the robustness of the primary outcome, we will conduct a mixed‐effects linear regression model with site as a random effect, adjusting for other interventions. Continuous outcomes assessed at multiple time points (such as blood markers of cardiac or kidney injury) will be analyzed using linear mixed models, including a random intercept for each patient and a random effect for site. The treatment‐by‐time interaction will indicate whether the endpoint changes differently over time in the intervention versus control group. We will report the results as relative percentage differences and use logarithmic transformation to approximate a normal distribution as appropriate and bootstrapping in case of deviations from distributional assumptions.

Categorical endpoints (e.g., unconscious at 96 h, number of CAM‐ICU positive patients) will be analyzed by the chi‐squared or Fisher's exact test, as appropriate, and presented as proportions with risk ratios and 95% CI. For the adjusted analysis we will apply mixed‐effects logistic regression models with site as a random effect and other interventions as fixed effect.

Ordinal endpoints (e.g., CPC and mRS scores) will be analyzed using proportional odds models with robust standard errors clustered by site and other interventions as fixed effects. Results will be reported as odds ratios with 95% CI.

#### 2.7.4 Analysis of Survival Data

The secondary endpoint survival at 90‐days will be evaluated using log‐binomial regression to estimate the relative risk of 90‐day mortality, defined as the proportion of patients who die from any cause between randomization and day 90. Additionally, Kaplan–Meier curves for each allocation group will be estimated, graphically displayed, and compared by the log‐rank test. Cox proportional hazard models will be applied to assess differences in time to death between treatment groups, estimating hazard ratios (HR) with 95% confidence intervals. These models will sequentially be adjusted for interaction between treatment allocations, and each of the following variables: sex, age, time to ROSC, lactate level upon admission, shockable primary rhythm, witnessed cardiac arrest, bystander performed cardiopulmonary resuscitation, presence of STEMI in the admission ECG. Assumptions will be assessed using Schoenfeld residuals and log–log plots. Furthermore, models considering cause of death (cardiovascular, neurological, multi‐organ failure) as competing risks will be performed by estimating the cumulative incidence for each type of death by the Nelson‐Aalen estimator.

### 2.8 Sub‐Studies

Predefined sub‐studies at the different sites have been included as part of the trial. At Aarhus University Hospital (from May 2025) and Copenhagen University Hospital (from July 2025), patients enrolled will have the following non‐invasive neuromonitoring modalities performed at pre‐defined time‐points 0–6 h, 24 ± 4 h, 48 ± 4 h and 66–72 h after randomization if they are still unconscious: Transcranial doppler ultrasound, optic nerve sheath diameter measured by ultrasound, automated pupillometry, and continuous EEG. The sub‐studies investigate early neuromonitoring predictors of successful wake‐up call and repeated neuromonitoring measurements across the wake‐up‐call intervention arms.

### 2.9 Biobank

A biobank containing blood drawn at several time points has been established. Neuron‐specific enolase is the only predefined blood‐based biomarker of outcome in the trial; however, further sub‐studies will investigate other markers of brain injury, as well as inflammation, and the trial biobank will further contribute to investigations on new markers of potential prognostic importance and insights to disease mechanisms.

### 2.10 Publication Plan

The findings from each of the four DANOHCA interventions will be reported as separate manuscripts submitted to peer‐reviewed international journals, and all results—whether beneficial, neutral, or harmful—will be published. In accordance with the EU Clinical Trials Regulation, summary results will also be publicly accessible through the EU Clinical Trials Information System (CTIS) and automatically posted on the EU Clinical Trials Register within 1 year of the end of the trial.

### 2.11 Safety and Adverse Events

#### 2.11.1 Adverse Events

Comatose OHCA patients requiring post‐resuscitation intensive care have a high risk of morbidity and mortality and thus, a high probability of adverse events (AE). Therefore, the following events will not be considered an AE in the present study, as they are frequently occurring in OHCA patients during ICU stay: myocardial infarction, minor bleeding, metabolic disorder, and other biochemical abnormalities.

AEs reported in the eCRF are categorized according to the following definitions: Major bleeding, infection, renal impairment, electrolyte disorders, metabolic disorder, cardiac, respiratory, seizures, extra pyramidal side effects, skin, withdrawal of life sustaining therapies and subsequent death as well as other adverse events potentially related to the intervention. For each AE reporting, there will be an additional question regarding whether there has been a serious adverse event (SAE). An example relevant to the early wake‐up call intervention would be an unplanned extubation requiring urgent reintubation, which will be captured as an SAE if it meets the standard seriousness criteria.

#### 2.11.2 Monitoring of Adverse Events and Serious Adverse Events

Patients are monitored for the development of an AE for 30 days after randomization or until hospital discharge if this occurs later than 30 days. A pre‐specified form in the electronic CRF allows for daily recording of AE during ICU stay. AE occurring after discharge from the ICU and within 30 days from randomization will be evaluated at the time of ambulatory follow‐up (scheduled for 3–6 months after cardiac arrest). AE will only be reported to the trial sponsor if it can be classified as a serious adverse event (SAE) with a suspected direct relation to the trial intervention. Once a year, all SAEs occurring at the trial centers will be submitted and a safety report of all trial patients will be submitted to the competent authorities as required. All SAEs will be reported as endpoint measures in the final trial report.

An Independent Data Safety Monitoring Board oversaw the trial conduct and an interim analysis with de‐identified data for safety was performed by 500 patients out of the preplanned 1000 patients. For this analysis, safety was assessed based on the respective primary endpoints for the four interventions and the occurrence of the protocol specified categories of serious adverse events. The interim analysis did not result in any remarks and the trial proceeded unchanged.

### 2.12 Trial Status and Timeline

Randomization began in June 2023, and patient inclusion is currently proceeding according to the expected timeline, with the last patients of a total of 1000 patients expected to be included in July 2026. Follow‐up is expected to be completed in October 2026.

## 3 Discussion

The DANOHCA trial will evaluate four potentially beneficial interventions in patients who remain comatose after resuscitation from OHCA aiming to reduce the morbidity and mortality associated with the post‐cardiac arrest syndrome, including an early wake‐up call strategy.

### 3.1 Rationale for Sedation After Resuscitation From OHCA

Sedation and mechanical ventilation have long been integral to early post–cardiac arrest care, primarily to facilitate temperature control, limit shivering, and ensure patient–ventilator synchrony. Historically, targeted hypothermia at 32°C–34°C required deep and sustained sedation, and clinical routines evolved accordingly [25, 26]. With the shift toward active normothermia and fever prevention, prolonged deep sedation is no longer required solely to prevent shivering; yet sedation practices have largely remained unchanged. This creates clinical equipoise regarding the optimal timing of sedation interruption and awakening after resuscitation from OHCA.

Data from non–cardiac arrest ICU populations further support this equipoise. A systematic review of clinical trials in critically ill patients could not demonstrate the level of sedation's impact on neither the risk of death, delirium, or serious adverse events [27]. However, the Danish NONSEDA trial, which compared a strategy of no continuous sedation with light sedation in mechanically ventilated patients, found no overall difference in mortality. Nonetheless, a numerically 5.4% higher mortality in the non‐sedation group was observed. Although not definitive, these findings raise important safety considerations regarding aggressive termination of sedation [17]. Extrapolation to the post‐cardiac arrest population should be made cautiously, as patients resuscitated from OHCA often present with additional challenges, including hypoxic–ischemic brain injury and hemodynamic instability, which may increase vulnerability to premature awakening. Taken together, these data reinforce the uncertainty regarding optimal sedation strategies and underscore the need for randomized trials specifically targeting this population.

An important consideration is the subgroup of OHCA patients with more severe hypoxic–ischemic brain injury, in whom epileptiform activity or seizures may occur early after cardiac arrest. In such patients, sedation interruption could theoretically be harmful by unmasking or exacerbating seizure activity. However, evidence supporting a protective effect of deeper or prolonged sedation is limited. The TELSTAR trial found no improvement in neurological outcome with aggressive antiseizure treatment that included higher doses of sedative agents [28].

Early awakening may allow more timely neurological assessment, earlier extubation, and reduced exposure to mechanical ventilation, potentially shortening intensive care unit and hospital length of stay. Conversely, premature sedation interruption could theoretically increase the risk of agitation, self‐extubation, hemodynamic instability, or re‐intubation [12, 29, 30]. Robust evidence addressing this balance is lacking. The present comparison within the DANOHCA trial is designed to evaluate whether a standardized early wake‐up call strategy is feasible and safe in contemporary post–cardiac arrest care and whether it improves patient‐centered recovery.

### 3.2 Potential Consequences of the DANOHCA Trial

If an early wake‐up strategy improves days alive outside of hospital without compromising safety, the findings may support a shift toward earlier sedation interruption and extubation assessment in selected comatose survivors of OHCA. Such a change could reduce exposure to invasive ventilation, decrease resource utilization, and accelerate recovery without adversely affecting neurological outcomes.

Conversely, if no benefit is observed or if safety concerns are identified, the results will provide important reassurance regarding the current practice of delayed awakening and inform clinicians about the risks and limitations of earlier sedation interruption. In this setting, safety concerns refer to extubation requiring urgent reintubation, respiratory compromise, hemodynamic instability, arrhythmias, and seizure activity. Regardless of outcome, the trial will contribute high‐quality evidence to guide sedation and ventilation strategies in a population where practice is highly variable.

Additionally, a recently published protocol for the STEPCARE SED‐CARE trial outlines a randomized comparison of sedation strategies in comatose survivors of OHCA, closely paralleling the present study. Although both trials address sedation and awakening in the OHCA population, differences in intervention design particularly regarding sedation targets and timing of wake‐up call, as well as outcome measures are expected to yield complementary insights. Collectively, these large‐scale trials, with a combined sample size exceeding 4.500 patients, will provide robust evidence to inform sedation strategies in post–cardiac arrest care [31].

### 3.3 Strengths and Limitations

The strengths of this study include its randomized design, multicenter setting, pragmatic integration into routine intensive care practice, and use of a patient‐centered primary outcome that captures both survival and time spent outside hospital. The factorial design allows efficient evaluation of multiple interventions within the same trial population. The trial is conducted within a single national healthcare system, which may affect generalizability to settings with different post–cardiac arrest care structures. On the other hand, the study population consists of all OHCA patients with a presumed cardiac cause in Denmark during the study period which will increase generalizability compared to studies based on more heterogeneous populations.

Limitations include the open‐label nature of the wake‐up call intervention, which is unavoidable given the intervention's characteristics, and the potential for protocol deviations driven by clinical judgment. Finally, although the study is powered for the primary outcome, it may not detect rare adverse events related to early awakening.

## 4 Conclusion

The early versus late wake‐up call comparison within the DANOHCA trial addresses a fundamental and unresolved aspect of post–cardiac arrest intensive care: the optimal timing of sedation interruption and assessment for extubation in comatose survivors of OHCA. By evaluating a protocolized early wake‐up strategy against current practice using a pragmatic, randomized design and a patient‐centered primary outcome, this trial aims to generate robust evidence to inform future clinical practice.

Regardless of the direction of effect, the results of this comparison will clarify the feasibility and safety of earlier awakening in contemporary normothermia‐based post–cardiac arrest care and contribute important data to guide sedation and ventilation strategies in this high‐risk population.

## Author Contributions

A.M.G. conceived the wake‐up call intervention, coordinated protocol development, drafted the manuscript, and contributed substantially to the study design, methodology, and interpretation of the scientific rationale. A.M.G., J.K., J.B.A., B.S.R., C.H., M.A.S.M., L.P.K.A., H.S., Si.M., S.M., C.T., S.C., and J.E.M. contributed substantially to the conception and overall design of the DANOHCA trial and critically revised the manuscript for important intellectual content. S.M. contributed to the statistical design and statistical analysis plan All authors contributed to the refinement of the protocol, approved the final version of the manuscript, and agree to be accountable for all aspects of the work.

## Funding

This work was supported by the Novo Nordisk Foundation (NNF22OC0079649), the Danish Heart Association (2023‐12401), and the Central Denmark Region Health Research Foundation.

## Conflicts of Interest

Christian Hassager reports grants from the Lundbeck Foundation, the Novo Nordisk Foundation, and The Danish Heart Foundation; and speaker's honorarium from Abiomed and BD. Jacob Eifer Moeller reports institutional grants from the Novo Nordisk Foundation and Abiomed; speakers’ fee from Abbott and Abiomed; and advisory board from Boston Scientific and Magenta. All outside the submitted work. Jesper Kjaergaard reports grant from Novo Nordisk Foundation (current trial) and Sundhedsdonationer (another research). Speakers fee from Vicare A/S. The remaining authors declare no conflicts of interest.

## Data Availability

The data that support the findings of this study are available from the corresponding author upon reasonable request.

## References

[1] E. Baldi , J. Wnent , M. L. Caputo , et al., “European Resuscitation Council Guidelines 2025 Epidemiology in Resuscitation,” Resuscitation 215, no. 1 (2025): 110733, 10.1016/J.RESUSCITATION.2025.110733. 41117565

[2] E. Dillenbeck , L. Svensson , A. Rawshani , et al., “Neurologic Recovery at Discharge and Long‐Term Survival After Cardiac Arrest,” JAMA Network Open 7, no. 10 (2024): e2439196, 10.1001/JAMANETWORKOPEN.2024.39196. 39392629 PMC11581594

[3] J. T. Gräsner , J. Wnent , R. Lefering , et al., “European Registry of Cardiac Arrest Study THREE (EuReCa‐ THREE)—EMS Response Time Influence on Outcome in Europe,” Resuscitation 223 (2025): 110704, 10.1016/J.RESUSCITATION.2025.110704. 40633749

[4] J. P. Nolan , C. Sandroni , A. Cariou , et al., “European Resuscitation Council and European Society of Intensive Care Medicine Guidelines 2025: Post‐Resuscitation Care,” Intensive Care Medicine 51, no. 12 (2025): 2213–2288, 10.1007/s00134-025-08117-3. 41123621

[5] C. Sandroni , S. D'Arrigo , S. Cacciola , et al., “Prediction of Poor Neurological Outcome in Comatose Survivors of Cardiac Arrest: A Systematic Review,” Intensive Care Medicine 46, no. 10 (2020): 1803–1851, 10.1007/S00134-020-06198-W. 32915254 PMC7527362

[6] V. Lemiale , F. Dumas , N. Mongardon , et al., “Intensive Care Unit Mortality After Cardiac Arrest: The Relative Contribution of Shock and Brain Injury in a Large Cohort,” Intensive Care Medicine 39, no. 11 (2013): 1972–1980, 10.1007/S00134-013-3043-4. 23942856

[7] I. Dragancea , M. Rundgren , E. Englund , H. Friberg , and T. Cronberg , “The Influence of Induced Hypothermia and Delayed Prognostication on the Mode of Death After Cardiac Arrest,” Resuscitation 84, no. 3 (2013): 337–342, 10.1016/J.RESUSCITATION.2012.09.015. 23000363

[8] T. Cronberg , G. Lilja , J. Horn , et al., “Neurologic Function and Health‐Related Quality of Life in Patients Following Targeted Temperature Management at 33°C vs 36°C After Out‐Of‐Hospital Cardiac Arrest: A Randomized Clinical Trial,” JAMA Neurology 72, no. 6 (2015): 634–641, 10.1001/JAMANEUROL.2015.0169. 25844993

[9] Y. Shehabi , R. Bellomo , M. C. Reade , et al., “Early Intensive Care Sedation Predicts Long‐Term Mortality in Ventilated Critically Ill Patients,” American Journal of Respiratory and Critical Care Medicine 186, no. 8 (2012): 724–731, 10.1164/RCCM.201203-0522OC. 22859526

[10] P. P. Pandharipande , T. D. Girard , J. C. Jackson , et al., “Long‐Term Cognitive Impairment After Critical Illness,” New England Journal of Medicine 369, no. 14 (2013): 1306–1316, 10.1056/NEJMOA1301372. 24088092 PMC3922401

[11] Y. Shehabi , L. Chan , S. Kadiman , et al., “Sedation Depth and Long‐Term Mortality in Mechanically Ventilated Critically Ill Adults: A Prospective Longitudinal Multicentre Cohort Study,” Intensive Care Medicine 39, no. 5 (2013): 910–918, 10.1007/S00134-013-2830-2. 23344834 PMC3625407

[12] J. W. Devlin , Y. Skrobik , C. Gélinas , et al., “Clinical Practice Guidelines for the Prevention and Management of Pain, Agitation/Sedation, Delirium, Immobility, and Sleep Disruption in Adult Patients in the ICU,” Critical Care Medicine 46, no. 9 (2018): E825–E873, 10.1097/CCM.0000000000003299. 30113379

[13] R. J. Stephens , M. R. Dettmer , B. W. Roberts , et al., “Practice Patterns and Outcomes Associated With Early Sedation Depth in Mechanically Ventilated Patients: A Systematic Review and Meta‐Analysis,” Critical Care Medicine 46, no. 3 (2018): 471–479, 10.1097/CCM.0000000000002885. 29227367 PMC5825247

[14] N. Watson , G. Karamasis , K. Stathogiannis , et al., “Feasibility of Early Waking Cardiac Arrest Patients Whilst Receiving Therapeutic Hypothermia: The Therapeutic Hypothermia and Early Waking (THAW) Trial,” Resuscitation 171, no. 1 (2022): 114–120, 10.1016/J.RESUSCITATION.2021.11.031. 34848275

[15] D. L. Staudacher , L. Heine , J. Rilinger , et al., “Impact of Sedation Depth on Neurological Outcome in Post‐Cardiac Arrest Patients ‐ A Retrospective Cohort Study,” Resuscitation 205 (2024): 110456, 10.1016/J.RESUSCITATION.2024.110456. 39631495

[16] A. Ceric , J. Dankiewicz , T. Cronberg , et al., “Sedation and Analgesia in Post‐Cardiac Arrest Care: A Post Hoc Analysis of the TTM2 Trial,” Critical Care 29, no. 1 (2025): 247, 10.1186/S13054-025-05461-0. 40528173 PMC12175406

[17] H. T. Olsen , H. K. Nedergaard , T. Strøm , et al., “Nonsedation or Light Sedation in Critically Ill, Mechanically Ventilated Patients,” New England Journal of Medicine 382, no. 12 (2020): 1103–1111, 10.1056/NEJMOA1906759/SUPPL_FILE/NEJMOA1906759_DATA-SHARING.PDF. 32068366

[18] J. Dankiewicz , T. Cronberg , G. Lilja , et al., “Hypothermia Versus Normothermia After Out‐Of‐Hospital Cardiac Arrest,” New England Journal of Medicine 384, no. 24 (2021): 2283–2294, 10.1056/NEJMOA2100591. 34133859

[19] DANOHCA , Protocol (01.09.25), 2025.

[20] C. Sandroni , S. D'Arrigo , and J. P. Nolan , “Prognostication After Cardiac Arrest,” Critical Care 22 (2018): 1, 10.1186/S13054-018-2060-7. 29871657 PMC5989415

[21] P. Stammet , O. Collignon , C. Hassager , et al., “Neuron‐Specific Enolase as a Predictor of Death or Poor Neurological Outcome After Out‐Of‐Hospital Cardiac Arrest and Targeted Temperature Management at 33°C and 36°C,” Journal of the American College of Cardiology 65, no. 19 (2015): 2104–2114, 10.1016/J.JACC.2015.03.538. 25975474

[22] E. W. Ely , A. Shintani , B. Truman , et al., “Delirium as a Predictor of Mortality in Mechanically Ventilated Patients in the Intensive Care Unit,” JAMA 291, no. 14 (2004): 1753–1762, 10.1001/JAMA.291.14.1753. 15082703

[23] B. Jennett and M. Bond , “Assessment of Outcome After Severe Brain Damage. A Practical Scale,” Lancet 305, no. 7905 (1975): 480–484, 10.1016/S0140-6736(75)92830-5. 46957

[24] J. Rankin , “Cerebral Vascular Accidents in Patients Over the Age of 60. II. Prognosis,” Scottish Medial Journal 2, no. 5 (1957): 200–215, 10.1177/003693305700200504. 13432835

[25] J. P. Nolan , R. W. Neumar , C. Adrie , et al., “Post‐Cardiac Arrest Syndrome: Epidemiology, Pathophysiology, Treatment, and Prognostication. A Scientific Statement From the International Liaison Committee on Resuscitation; the American Heart Association Emergency Cardiovascular Care Committee; the Council on Cardiovascular Surgery and Anesthesia; the Council on Cardiopulmonary, Perioperative, and Critical Care; the Council on Clinical Cardiology; the Council on Stroke,” Resuscitation 79, no. 3 (2008): 350–379, 10.1016/J.RESUSCITATION.2008.09.017. 18963350

[26] C. Sandroni , J. P. Nolan , L. W. Andersen , et al., “ERC‐ESICM Guidelines on Temperature Control After Cardiac Arrest in Adults,” Intensive Care Medicine 48, no. 3 (2022): 261–269, 10.1007/S00134-022-06620-5. 35089409

[27] A. Ceric , J. Holgersson , T. L. May , et al., “Effect of Level of Sedation on Outcomes in Critically Ill Adult Patients: A Systematic Review of Clinical Trials With Meta‐Analysis and Trial Sequential Analysis,” EClinicalMedicine 71 (2024): 71, 10.1016/J.ECLINM.2024.102569. PMC10990717 38572080

[28] B. J. Ruijter , H. M. Keijzer , M. C. Tjepkema‐Cloostermans , et al., “Treating Rhythmic and Periodic EEG Patterns in Comatose Survivors of Cardiac Arrest,” New England Journal of Medicine 386, no. 8 (2022): 724–734, 10.1056/NEJMOA2115998. 35196426

[29] J. P. Kress , A. S. Pohlman , M. F. O'Connor , and J. B. Hall , “Daily Interruption of Sedative Infusions in Critically Ill Patients Undergoing Mechanical Ventilation,” New England Journal of Medicine 342, no. 20 (2000): 1471–1477, 10.1056/NEJM200005183422002. 10816184

[30] T. D. Girard , J. P. Kress , B. D. Fuchs , et al., “Efficacy and Safety of a Paired Sedation and Ventilator Weaning Protocol for Mechanically Ventilated Patients in Intensive Care (Awakening and Breathing Controlled Trial): A Randomised Controlled Trial,” Lancet 371, no. 9607 (2008): 126–134, 10.1016/S0140-6736(08)60105-1. 18191684

[31] A. Ceric , J. Dankiewicz , J. Hästbacka , et al., “Continuous Deep Sedation Versus Minimal Sedation After Cardiac Arrest and Resuscitation (SED‐CARE): A Protocol for a Randomized Clinical Trial,” Acta Anaesthesiologica Scandinavica 69, no. 5 (2025): e70022, 10.1111/AAS.70022. 40178107 PMC11967157

